# Dermal tattoo biosensors

**DOI:** 10.1007/s00105-023-05195-6

**Published:** 2023-07-14

**Authors:** Kailas Dhond, Yubing Hu, Ali K. Yetisen

**Affiliations:** Department of Chemical Engineering, Imperial College London, SW7 2AZ London, UK

## Rethinking point-of-care testing

Healthcare systems have historically been designed for the treatment of acute problems, and few practices for continuous long-term monitoring are in place. There is an urgent need for a more proactive approach to long-term disease [1]. Dermal tattoo biosensors are emerging platforms that enable continuous biomarker monitoring for optimization of disease management and treatment. Tattoo biosensors will reduce medical costs and revolutionise point-of-care diagnostics.

## Skin as a diagnostic platform

The skin is the largest organ in the body and comprises the epidermis, dermis, and hypodermis layers. The dermis is the middle layer of skin, and primarily contains fibroblasts, macrophages, and adipocytes. It is the layer of skin that contains the most interstitial fluid (ISF). The ISF is the fluid that encompasses cells in the tissue, supplying them with nutrients and helping diffuse out cellular waste [2]. The ISF contains many biomarkers that are also found in blood, and its closer proximity to the skin allows one to access this fluid without the penetration depths required with blood. A variety of methods exist for extracting ISF for diagnostic purposes, and these methods provide an easily accessible and low-cost means of analysing biomarkers within the body. Procedures such as wick insertion for ISF absorption, vacuum suction blisters, microdialysis via membrane filtration, and reverse iontophoresis can be used to obtain interstitial fluid. However, they are time consuming, are often incapable of achieving adequate volumes, and can distort concentrations of ions and metabolites. Moreover, extracting interstitial fluid is limiting in that it only enables measurement of metabolites at discrete time points [2].

A newly emerging concept in medicine involves using non-invasive or minimally invasive biosensors to continuously monitor the body’s biomarker concentrations. Biosensors enable detection of biomarkers within the body, and entail a specific target analyte and receptor, which upon binding produce a physicochemical signal [3]. These biosensors would eliminate the need to extract bodily fluids for measurement. The ideal biosensor would have access to a vast number of biomarkers and be able to function long term. Continuous sweat and tear sensors to monitor biomarkers have been developed, although their efficacy is held back by the lack of alignment with blood biomarker concentration and poor calibration retention [4]. Dermal sensors monitoring the ISF can provide a means for continuous monitoring of a wide array of biomarkers. Electrochemical sensors are the most common type of dermal sensor. Interstitial fluid-sensing microneedle-based and reverse-iontophoresis electrochemical sensors have been explored for levodopa and glucose monitoring, respectively. Although they can achieve high sensitivity, electrochemical sensors require electrodes, which confer poor biocompatibility and limit their lifespan [5]. Hydrogels are commonly used in the design of sensors due to their hydrophilic nature and biocompatibility; however, they tend to be more expensive and not durable enough for long-term monitoring [6]. There is an urgent need to develop long-lasting and biocompatible sensors for chronic disease monitoring.

Dermal biosensors delivered through the medium of a tattoo present a novel method for continuous monitoring. Tattoos are an ancient form of body art that date back thousands of years. An analysis of a Tyrolean Iceman, whose body was estimated to be 5300 years old, found over 61 tattoos consisting of clusters of lines between 10 and 40 mm in length. The close proximity of the tattoos to joints popularised the theory that these ancient tattoos had medicinal effects, possibly constituting a primitive form of acupuncture [7]. Today, tattoos are primarily used for cosmetic effect, but can be used medically to help camouflage skin blemishes, such as in the case of vitiligo [8]. Tattooing is predominantly done with a handheld tattoo gun, which injects pigment granules into the dermis layer of the skin. The capacity for tattoos to be employed as a medical tool for continuous monitoring is largely unexplored, and could provide advantages over traditional wearable sensors. Tattoos provide easy access to the biomarker-rich ISF and can stay in the dermis for extended periods. Moreover, tattoos are becoming increasingly more prevalent in Western culture, with 24% of Americans between adolescence and 60 years of age and over 100 million Europeans having at least one tattoo [9]. Given the rise in tattoo prevalence, and the predicted market value of wearable sensors by 2025 to be 2.86 billion U.S. dollars, the pathway for wearable tattoo sensors is promising [10].

In recent years, chromogenic biosensing tattoo inks have been developed to detect changes in pH, glucose, and albumin in ISF over a normal human physiological range [4]. Using ex vivo porcine skin as a surrogate for human skin, Yetisen et al. were able to validate a gradual colour change of the porcine skin from yellow to blue over a pH range of 5.0–9.0. Moreover, their glucose colorimetric tattoo sensor showed colorimetric changes from yellow to dark green within 30 s, allowing a near instantaneous reading. The researchers also developed an albumin sensor using the chromogenic substance 3′,3′′,5′,5′′-tetrachlorophenol‑3,4,5,6-tetrabromosulfophthalein, and described a visible change from yellow to green with increasing levels of albumin [4]. These optical tattoo biosensors present a novel way to continuously monitor crucial biomarkers without the need for electrical power.

Tattoo biosensors can be interpreted via direct observation of colorimetric changes by the naked eye, or viewed through an intermediary. Jiang et al. used tattoo fluorescent biosensors in conjunction with a battery powered light-emitting diode (LED) readout device attached to the skin. The tattoo sensor made it possible to track hydrogen, potassium, and sodium concentrations [11]. Each analyte had an optical filter and LED with an appropriate excitation wavelength. A smartphone camera was used to take pictures of the readout device, and enabled quantitative analysis of hydrogen, potassium, and sodium ions [11]. Moreover, the readout device improved the accuracy of the device, as readings were less influenced by variability in ambient lighting conditions.

Chronic diseases, primarily cardiovascular or respiratory diseases, cancer, and diabetes, are the cause of 71% of all deaths annually [12]. Dermal tattoo sensors can provide information necessary to improve the quality of treatment and potentially aid in earlier detection of these diseases. By 2030, The Global Strategy on Human Resources for Health predicts a global shortage of 18 million healthcare staff [13]. Dermal tattoo biosensors will be vital in the future as the global population increases and healthcare disparities worsen. People will need to take more responsibility for monitoring their own health and adopt a more proactive approach. Moreover, healthcare shortages will disproportionately affect those in lower-income countries, especially in Africa [13]. Low-cost biosensors that can be used with minimal training and require no electrical power source are the perfect solution to this crisis.

Dermal tattoo sensors have a long-lifespan potential because of the macrophages and fibroblasts that engulf any dead cells, thus allowing the tattoo sensors to remain in the dermis for long periods without being metabolised [11]. Although current progress in dermal tattoo sensors shows great promise, further work is required before they can be implemented in a clinical setting. Possible complications could exist regarding infections or allergies as a result of tattoo inks, with reported rates of tattoo-related skin infections ranging from 1% to 5% [8, 9]. Additionally, the sterility of commercially available inks is poorly regulated, and commonly contains contaminants [14]. Tattoo inks also contain carcinogenic polycyclic aromatic carbons and toxic heavy metals such as mercury, cadmium, cobalt, and chromium. Removal of the tattoo biosensors also poses an issue, as current methods for tattoo removal typically require multiple treatments over several months, and can result in scarring, altered pigmentation, and blisters [8]. There remains to be any clinical trials evaluating the safety and efficacy of tattoo biosensors in humans over extended periods. He et al. were able to monitor pH and temperature in rabbit skin for 4 days using a colorimetric tattoo sensor, but an ideal sensor would be able to work for several months [15]. The sensors are currently restricted in their efficacy due to the small number of biomarkers that have been successfully measured, and due to our limited understanding of analyte correlation between ISF and blood concentrations [10]. Moreover, the accuracy of tattoo sensors relies on a consistent tattoo depth maintained in the skin in order for a well-calibrated and repeatable tattoo reading to be made [15].

## Practical conclusion

- Tattoo sensors are ushering in a new era of injectable sensors with possibilities for high biocompatibility and ease of use.
- Currently, most tattoo biosensors are low cost and only require a smartphone for analyte quantification, making them widely accessible to lower-income parts of the world.
- Moreover, their potential for continuous multiplexed biomarker detection could help equip healthcare systems with the necessary tools to better deal with long-term illnesses.

## Declarations

For this article no studies with human participants or animals were performed by any of the authors. All studies mentioned were in accordance with the ethical standards indicated in each case.
